# Identification of Mendelian inconsistencies between SNP and pedigree information of sibs

**DOI:** 10.1186/1297-9686-43-34

**Published:** 2011-10-11

**Authors:** Mario PL Calus, Han A Mulder, John WM Bastiaansen

**Affiliations:** 1 Animal Breeding and Genomics Centre, Wageningen UR Livestock Research, 8200 AB Lelystad, The Netherlands; 2 Animal Breeding and Genomics Centre, Wageningen University, 6709 PG Wageningen, The Netherlands

## Abstract

**Background:**

Using SNP genotypes to apply genomic selection in breeding programs is becoming common practice. Tools to edit and check the quality of genotype data are required. Checking for Mendelian inconsistencies makes it possible to identify animals for which pedigree information and genotype information are not in agreement.

**Methods:**

Straightforward tests to detect Mendelian inconsistencies exist that count the number of opposing homozygous marker (e.g. SNP) genotypes between parent and offspring (PAR-OFF). Here, we develop two tests to identify Mendelian inconsistencies between sibs. The first test counts SNP with opposing homozygous genotypes between sib pairs (SIBCOUNT). The second test compares pedigree and SNP-based relationships (SIBREL). All tests iteratively remove animals based on decreasing numbers of inconsistent parents and offspring or sibs. The PAR-OFF test, followed by either SIB test, was applied to a dataset comprising 2,078 genotyped cows and 211 genotyped sires. Theoretical expectations for distributions of test statistics of all three tests were calculated and compared to empirically derived values. Type I and II error rates were calculated after applying the tests to the edited data, while Mendelian inconsistencies were introduced by permuting pedigree against genotype data for various proportions of animals.

**Results:**

Both SIB tests identified animal pairs for which pedigree and genomic relationships could be considered as inconsistent by visual inspection of a scatter plot of pairwise pedigree and SNP-based relationships. After removal of 235 animals with the PAR-OFF test, SIBCOUNT (SIBREL) identified 18 (22) additional inconsistent animals.

Seventeen animals were identified by both methods. The numbers of incorrectly deleted animals (Type I error), were equally low for both methods, while the numbers of incorrectly non-deleted animals (Type II error), were considerably higher for SIBREL compared to SIBCOUNT.

**Conclusions:**

Tests to remove Mendelian inconsistencies between sibs should be preceded by a test for parent-offspring inconsistencies. This parent-offspring test should not only consider parent-offspring pairs based on pedigree data, but also those based on SNP information. Both SIB tests could identify pairs of sibs with Mendelian inconsistencies. Based on type I and II error rates, counting opposing homozygotes between sibs (SIBCOUNT) appears slightly more precise than comparing genomic and pedigree relationships (SIBREL) to detect Mendelian inconsistencies between sibs.

## Background

Use of many SNP genotypes to apply genomic selection in breeding programs is becoming common practice. With the increasing importance of this new information source, the need for tools to edit and check the quality of this data increases as well. One of the common editing steps for marker (e.g. SNP) data, is to check for Mendelian inconsistencies [1]. A Mendelian inconsistency occurs when the genotype and pedigree data of two related animals are in disagreement. A clear example is when an animal is homozygous for one allele (e.g. AA), while its parent is homozygous for the other allele (e.g. CC), i.e. the two animals have 'opposing' homozygote genotypes [2]. This may result from an error in the recorded pedigree, from genotyping errors, or from mixing up DNA samples and in very rare cases from mutations. Checking for opposing homozygotes is a commonly used test for example for paternity testing e.g. [3].

Mendelian inconsistencies are usually identified by comparing the genotypes of one or both parents to the genotypes of their offspring. This comparison is straightforward, since it only involves checking for each locus whether one of the two alleles that the individual has could have been inherited from one of its parents. The expected number of inconsistencies between a genotyped parent-offspring pair and the variance of this expected number is very low when opposing homozygotes only result from genotyping errors [2]. When two related genotyped animals are separated by more than one meiosis, the expected number of SNP with opposing homozygotes is greater than zero, even in the absence of genotyping errors. The expected number of opposing homozygous genotypes is related to the additive genetic relationship between two animals, since this relationship is equivalent to the expected proportion of identical by descent shared genome [4,5]. The variance of the expected number of opposing homozygous genotypes, therefore, depends on the variance of the additive genetic relationship between two animals. The variance of relationships, in turn, was shown to depend on Mendelian sampling (i.e. the number of meiotic events between two animals) e.g. [6,7]. A common example, where an animal's closest genotyped relative is separated by more than one meiosis, is when the other animal is a grandparent or a sib. In breeding schemes, only sires may be genotyped, such that the closest genotyped relative on the dam side is a maternal grandsire [1]. One or more sibs may be the closest genotyped relative(s) when the common parent(s) of the animals are not genotyped. More specifically, breeding populations may contain many genotyped (large) full or half-sib families. Extended pedigrees among genotyped animals provide the opportunity to compare the genotype of an animal to genotypes of multiple relatives, but this also increases the complexity of the comparison [8]. An alternative approach, compared to counting opposing homozygotes, is to derive relationships between all animals twice, using either pedigree or SNP information. When plotting pedigree and SNP-based relationships against each other, inconsistencies can be detected by identifying pairs of relationships that do not match by visual inspection of the scatter plot [9]. When, for example, the pedigree information indicates that two animals are full-sibs, with a pedigree-based relationship ≥ 0.5, but the relationship based on the genotype information is close to zero, we can expect a pedigree or sample mis-identification. To allow routine use of this comparative approach, a documented set of rules that can be used in an algorithm is required.

Therefore, the objective of this paper was to develop and demonstrate two tests, both comprising a set of rules that allow for the fast identification of sibs with conflicting genotype and pedigree information. The first test identifies sibs for which the number of contrasting homozygous genotypes does not match the expectation. The second test identifies sibs for which the pedigree and genomic relationships do not match. The performance of both tests was demonstrated on a dairy cattle dataset comprising predominantly genotyped cows. In addition, we derived the theoretical expectations and variance of the number of inconsistencies for unrelated animals, half-sibs and full-sibs, using observed allele frequencies.

## Methods

In this study, we compared two statistical tests to detect inconsistencies between pedigree and genotype information of supposed sib pairs. In both tests, the data was first checked for inconsistent parent-offspring pairs. Animals that were inconsistent with a supposed parent or offspring were detected, and problematic animals were iteratively removed, as described directly hereafter.

### Detecting parent-offspring inconsistencies (PAR-OFF)

Parent-offspring inconsistencies were detected by considering all pairs of animals that were supposed parent-offspring based either on the pedigree or the SNP information. For each genotyped pair of animals that were parent-offspring according to the pedigree, the number of opposing homozygous loci was counted. Two animals have opposing homozygous loci when one animal is homozygous for one allele, and the other animal is homozygous for the other allele. The realized distribution of the number of opposing homozygotes was used to define the threshold for declaring a parent-offspring pair inconsistent. Based on this distribution, we also identified all pairs of animals that were not parent-offspring pairs according to the pedigree, but that had a number of opposing homozygotes smaller than the threshold used for PAR-OFF similar to Hayes [2]. To avoid testing monozygotic twins, included pairs had to have different genotypes for more loci than the threshold applied by PAR-OFF for the number of opposing homozygote loci. All parent-offspring pairs that were identified based only on the SNP information, were also declared inconsistent.

Inconsistent parent-offspring pairs were removed as follows.

1. Both animals from inconsistent parent-offspring pairs were removed when both animals in the pair had no other 1^st ^degree genotyped relatives.

2. If the parent was already removed, due to inconsistency with its genotyped parent(s), then the offspring was left in the data.

3. When a parent had multiple genotyped offspring, it was removed only if it was inconsistent with more than 80% of its offspring. In all other cases, the inconsistent offspring were removed.

After removing animals, locus-specific inconsistent genotypes of remaining parent-offspring pairs were set to missing for both animals. Then, the Beagle software [10] was used to impute all genotypes for SNP with a known position on the 29 autosomes, that were either set to missing due to remaining locus specific inconsistent genotypes, or that were missing because of genotyping failures.

### Detecting sib inconsistencies

An iterative approach was used to discard animals from the dataset that caused inconsistencies between pairs of sibs. In the first step, all inconsistent pairs were identified and in subsequent steps, the animal with the highest number of inconsistencies was iteratively removed from the dataset until no inconsistencies remained. Detection of inconsistent pairs of sibs was either based on differences between pedigree and genomic relationships (SIBREL), or on the number of opposing homozygous genotypes (SIBCOUNT) between them.

### SIBCOUNT: counting opposing homozygotes between sibs

For each pair of genotyped animals for which pedigree records indicated that they were unrelated (i.e. that they had a pedigree relationship equal to zero), half-sibs, or full-sibs, the number of opposing homozygous loci was counted. Empirical distributions of the number of opposing homozygous loci were used to define minimum thresholds for declaring inconsistent pairs of unrelated animals, half-sibs, and full-sibs. Animal pairs that had the same genotype for (almost) all loci were also identified. This last category was expected to contain pairs of monozygotic twins based on the SNP information, but may have been caused by samples being mixed up (e.g. allocating two samples of one animal to two different pedigree entries). In other datasets, this category could also include split embryos used in embryo transfer and clones from nuclear transfer.

The empirical distributions of the number of opposing homozygotes were also compared to theoretically predicted distributions. The latter may be used when the number of observed relationships in a population for a given class is too low to obtain a proper empirical distribution. The expected number of opposing homozygous loci between two half-sibs is equal to $\sum_{i=1}^{n}p_{i}^{2}q_{i}^{2}$, considering *n *bi-allelic loci with allele frequencies *p_i _*and *q_i_*. Likewise, the expected number of opposing homozygous loci between two full-sibs is $\sum_{i=1}^{n}\frac{1}{2}p_{i}^{2}q_{i}^{2}$, and between two unrelated animals this is $\sum_{i=1}^{n}2p_{i}^{2}q_{i}^{2}$. Derivations for these expected numbers of opposing homozygotes for all three categories, and the expected variance thereof, are given in Appendix A.

### SIBREL: comparing pedigree and genomic relationships between sibs

Empirical distributions of pedigree and genomic relationships were first compared to expected distributions of relationships, which were derived in Appendix B. An algorithm was developed to efficiently compare pedigree and genomic relationships to identify inconsistent sib pairs. This algorithm comprises the following main steps that are explained in more detail below:

1. calculate the pedigree relationship matrix for all genotyped animals with consideration of inbreeding using the complete pedigree information,

2. calculate the genomic relationship matrix for all genotyped animals using genotype information,

3. rescale the genomic relationship matrix such that the average genomic inbreeding coefficient is the same as in the pedigree relationship matrix,

4. empirically derive the threshold for inconsistent pairs of half- and full-sibs, by identifying differences (i.e. lack of overlap) between distributions for different relationship classes,

5. identify half- and full-sib pairs that are inconsistent based on the threshold defined under 4.

#### Calculation and scaling of relationships (step 1 to 3)

The pedigree-based relationship matrix **A **was calculated using the algorithm of Meuwissen and Luo [11]. Genomic relationships were calculated as described by VanRaden [12]:

$$G=\frac{{ZZ}^{\prime }}{2\sum p_{i}\left(1-p_{i}\right)}$$

where *p_i _*is the frequency of the second allele at locus *i*, and **Z **is an incidence matrix that stores the genotypes of all animals at all loci. **Z **is calculated as matrix **M - **2(*p_i _*- 0.5). Matrix **M **contains elements -1, 0, and 1 for the three possible genotypes, where 1 codes for the genotype that is homozygous for the second allele. Note that **G **contains identical-by-state relationships, rather than identical-by-descent relationships. This means that the generation in which the allele frequencies *p_i _*are calculated, is considered to be the base generation, assuming that animals in that generation are unrelated. One way to put **G **and **A **on the same scale, is to estimate *p_i _*for the considered base generation in **A **(i.e. the first generation of the available pedigree). For simplicity, *p_i _*were calculated across all genotyped animals, meaning that the current population is the base generation, which implies that the genomic relationships were somewhat underestimated. To deal with this underestimation, **G **was rescaled as follows. The pedigree inbreeding coefficient was calculated for all animals, and averaged (denoted as $\bar{f_{p}}$). The genomic inbreeding coefficients were assumed to be on average zero since **G **assumes that the current population forms the base generation.

Finally, G* was obtained as:

$$G^{*}=G\left(1-\bar{f_{p}}\right)+2\bar{f_{p}}J$$

where G* contains relationships relative to the same base as used in **A**, and **J **is a matrix of all 1's. This formula to adjust **G **comes from Wright's F-statistics [13].

Elements of G* were used in the comparison of genomic and pedigree relationships.

#### Identification of inconsistencies between pedigree and genomic half- and full-sib relationships (step 4 to 5)

First, all pairs of animals with a genomic relationship > 0.95 were identified. In the present dataset, only monozygotic twins could reach this relationship. Therefore, all such pairs that were not full-sibs according to the pedigree, were declared inconsistent.

Secondly, based on the pedigree, all pairs of genotyped half- and full-sibs were identified. A half-sib or full-sib pair of animals *i *and *j*, based on pedigree, was declared inconsistent, when

$$|G_{i,j}-A_{i_{,}j}|\;>\gamma$$

The threshold γ was chosen to be 0.2 for both half- and full-sibs, based on the empirical distribution of *G_i,j _*- *A_i,j_*, as shown in the results section.

### Removing animals that cause inconsistencies

After identification of inconsistent pairs in step one of methods SIBCOUNT and SIBREL, the removal of animals from the dataset in the subsequent steps was the same for both methods. For each animal, the number of inconsistent half-sibs and full-sibs was counted. Animals that caused inconsistencies were removed using the following steps:

1. count for each animal the number of relationships it has for which the pedigree and genomic information are inconsistent,

2. sort the animals based on descending number of inconsistent sibs,

3. remove the animal that causes the largest number of inconsistencies; if that animal has only one genotyped sib, both animals are removed; when there is more than one animal with the largest number of inconsistent pairs, remove the animal with the lowest number of genotyped sibs,

4. recalculate the total number of inconsistencies for all remaining animals by subtracting the number of inconsistencies associated with the animal that was removed; go back to step 1 and repeat until all inconsistencies are removed.

Step 1 is the application of SIBCOUNT or SIBREL. After step 1, steps 2-4 were performed iteratively. In each iteration, the animal contributing the highest number of inconsistent sib pairs was assumed to have a real mismatch between pedigree and SNP genotypes and was therefore removed.

### Comparing SIBCOUNT and SIBREL

The performance of the proposed SIBCOUNT and SIBREL tests was verified based on the type I and II error rates of declared inconsistencies. The type I error rate gives the proportion of false positive inconsistencies, i.e. animals that are deleted due to inconsistencies but for which differences between SNP and pedigree information are not due to errors in pedigree or genotype information. Note that in extreme cases, animals with many genotyping errors, i.e. animals with multiple loci having incorrect genotypes, may be deleted by either test. Applying a stringent threshold for the proportion of missing SNP genotypes, however, ensures detection and deletion of such animals in an earlier step. The type II error rate gives the proportion of false negative inconsistencies, i.e. animals that are not deleted because an inconsistency is not declared, while their SNP and/or pedigree information are incorrect.

The type I and II error rates were both investigated as follows. First, all inconsistent animals based either on SIBCOUNT or SIBREL were deleted. This was done, to ensure that no animals were left in the data that could be deleted by either test. Secondly, the pedigree information for randomly selected 1, 10 or 25% of the remaining animals was permuted against the SNP information. In this permutation step, the link between animal ID and SNP information was left unchanged, but the pedigree information (i.e. sire and dam ID) was randomly shuffled amongst the permuted animals. This permutation simulated a situation in which possibly existing sib relationships based on the pedigree were not supported by the genotype information and vice versa. Pedigree relationships between all pairs of animals were compared before and after permutation. For all animals with a permuted pedigree, we checked if there was at least one other animal that was either a half- or full-sib, both before and after permuting the pedigree information. Such animals were deleted in this replicate to make sure that permuted animals really had inconsistent SNP and pedigree information. Finally, the type I error rate was calculated as the proportion of animals that were removed although their pedigree was correct (i.e. not permuted) and the type II error rate as the proportion of animals not removed although their pedigree was permuted. This whole process was done twice, once preceded by the PAR-OFF test, and once without doing the PAR-OFF test. When the PAR-OFF test was performed, type I and II error rates for SIBCOUNT and SIBREL were calculated based on the permuted animals that were not removed by PAR-OFF. Average type I and II error rates were calculated across 50 replicates of the permutation.

### Data

In total, 2,359 animals with known pedigree were genotyped using the Illumina BovineSNP50 BeadChip (54,001 SNP; Illumina, San Diego, CA). This data comprised Holstein-Friesian cows from experimental farms in Ireland, the UK, the Netherlands and Sweden, as well as sires of some of the genotyped cows. The quality control criteria for selecting the final set of SNP were a call rate of > 95%, a GenCall score > 0.2, and a GenTrain score > 0.55 (Illumina descriptive statistics relating to genotype quality), a minor allele frequency greater than 1% for each country, and a lack of deviation from Hardy-Weinberg equilibrium based on a χ^2 ^less than 600 [1]. Seventy animals with greater than 5% missing SNP genotypes were removed. After these initial checks, the data contained 2,289 animals with SNP genotypes for 36,884 loci. SNP on the X chromosome were not used because males only carry one copy of the X chromosome. The remaining 36,884 SNP had either a known position on one of the 29 autosomes or were not mapped to a chromosome. This edited SNP dataset contained 2,078 cows, and 211 sires. Sires had between 1 and 62 genotyped daughters. In total, the data contained 891 genotyped mother-daughter pairs, 1,448 genotyped father-daughter pairs, and 508 animals without any genotyped parent.

## Results

### Distribution of opposing homozygotes

**Figure 1:**
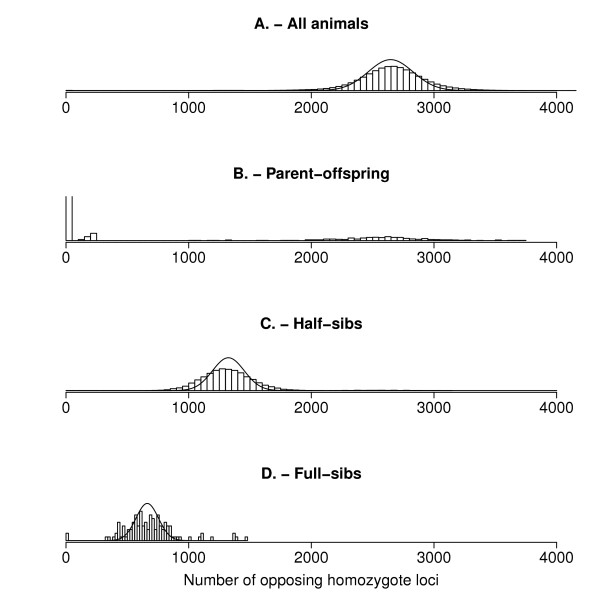
**Distributions of opposing homozygotes**. Empirical ('bars') and expected distributions (smoothed line) of the number of opposing homozygotes between pairs of: A. all animals, B. parent-offspring pairs, C. half-sibs and D. full-sibs.

The number of SNP loci with opposing homozygotes was first calculated between all pairs of animals (Figure 1A). Based on the distribution of the number of opposing homozygotes between parent-offspring pairs (Figure 1B), it was assumed that for all parent-offspring pairs with more than 250 opposing homozygous loci, a conflict existed between the pedigree and SNP data. Likewise, the threshold was considered to be 2,150 opposing homozygotes for half-sib pairs (Figure 1C), and 1,250 opposing homozygotes for full-sib pairs (Figure 1D). The expected mean and standard deviation of the number of opposing homozygotes were calculated for full-sibs, half-sibs, and unrelated animals (Table 1), and the corresponding distributions were plotted together with the empirical distributions (Figure 1). The expected distributions of the numbers of opposing homozygotes supported the empirically derived thresholds. This indicates that the derived formulas can be used to derive thresholds instead of the realized distributions, when there are too few values to empirically derive a threshold for a given class of relationships.

**Table 1:** Expected number of opposing homozygotes

	Expected number of opposing homozygotes^1^	
	Mean	Standard deviation
Full-sibs	661.37	91.29
Half-sibs	1322.75	129.10
Unrelated animals	2645.50	182.58

^1^Calculated based on the formulas presented in the Appendix

### Distribution of relationships

**Figure 2:**
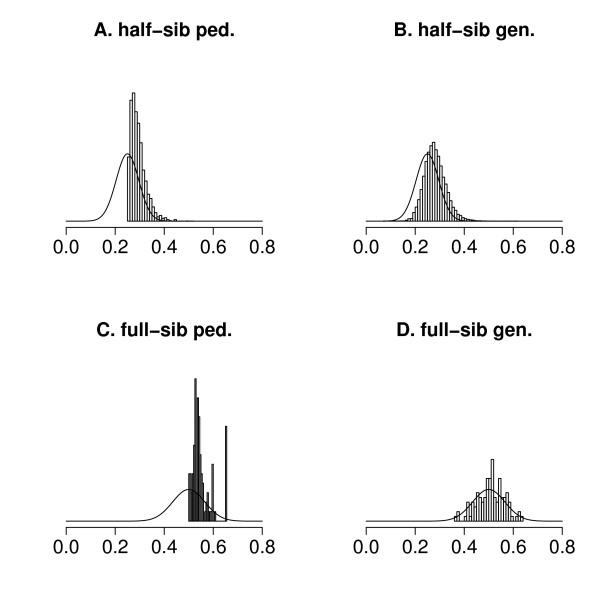
**Distributions of sib relationships**. Empirical ('bars') and expected distributions (smoothed line) of half-sib (A & B) and full-sib relationships (C & D), where empirical distributions are based on pedigree (A & C) or genomic information (B & D).

Relationships were calculated for pairs of half- and full-sibs, using either pedigree or SNP information (Figure 2 and Table 2). The expected mean relationships were smaller than the realized values (Table 2) because inbreeding was ignored in the expected values. Variances of genomic relationships were much closer to their expectations than variances of pedigree-based relationships (Figure 2 and Table 2), because genomic relationships track the real common portion on the genome.

**Table 2:** Expected and realized averages and SD of sib relationships

	**Half-sib**		**Full-sib**	
	**Average**	**SD**	**Average**	**SD**
	
Expected^1^	0.25	0.0464	0.5	0.0657
Pedigree (realized)	0.2937	0.0310	0.5504	0.0404
Genomic (realized)	0.2807	0.0524	0.5044	0.0726

^1^Average and SD of relationships are predicted ignoring inbreeding [23]

**Figure 3:**
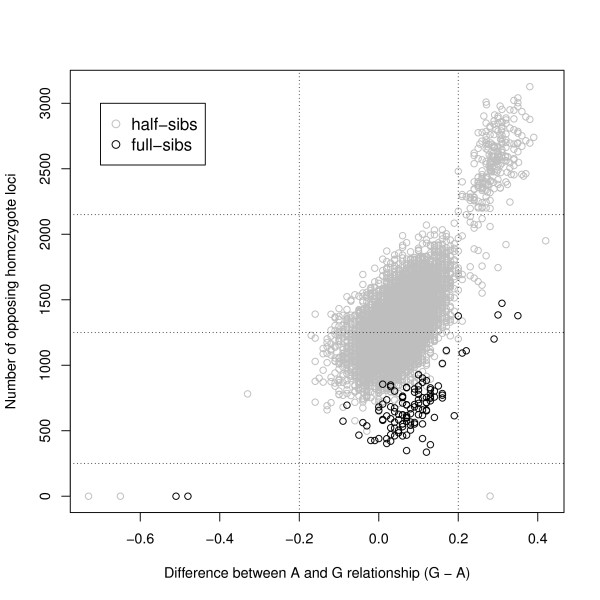
**Opposing homozygote loci versus difference between relationships**. Number of inconsistent SNP loci versus difference between pedigree and genomic relationship for half- and full-sibs.

To visualize the relationship between the number of opposing homozygotes and the difference between pedigree and genomic relationships, both these variables were plotted against each other across all half- and full-sib pairs (Figure 3). Based on Figure 3 a threshold of 0.2 was chosen for the difference in pedigree-based and genomic relationships to declare a pair of sibs inconsistent. We expected that this threshold would target largely the same pairs of sibs as the thresholds for Mendelian inconsistencies.

### Deleted animals due to inconsistencies

**Figure 4:**
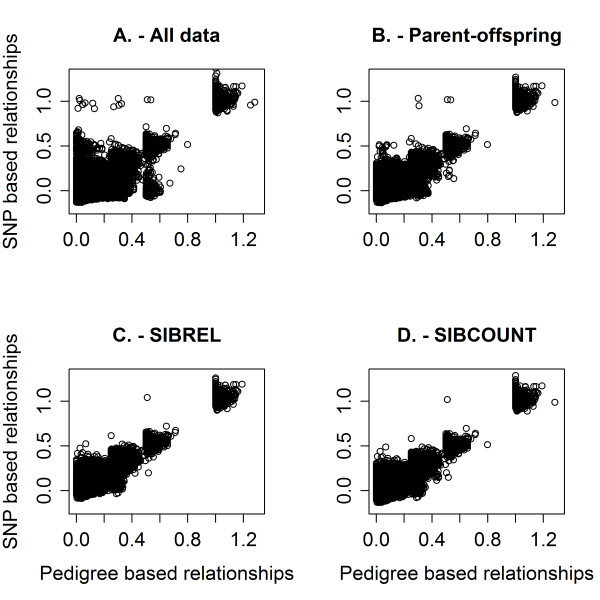
**Genomic versus pedigree relationships**. Before excluding any animals (A), after excluding animals based on parent-offspring Mendelian inconsistencies (B), after excluding animals based on sib relationships (SIBREL; C), and after excluding animals based on counted opposing homozygotes between sibs (SIBCOUNT; D).

Due to parent-offspring inconsistencies, 235 animals were removed from the data, of which 12 animals were part of a parent-offspring pair based on the SNP data but which was not supported by the pedigree data. Deleting the 235 animals with parent offspring inconsistencies removed many inconsistencies between the genomic and pedigree-based relationships, as can be seen by comparing Figures 4A (including all animals) and 4B (after removing those 235 inconsistent animals). Using the SIBCOUNT test, which detects inconsistenties between sibs by counting opposing homozygotes, 18 animals were removed. Using the SIBREL test, which detects inconsistencies between sibs by comparing pedigree and genomic sib relationships, 22 animals were removed. Seventeen animals were removed by both methods. In Table 3, the number of declared inconsistencies for each sib class is given for both methods. Results show that for both methods, an inconsistency in half-sib relationships was the main reason to remove animals. The numbers of declared inconsistencies were very similar for the two methods.

**Table 3:** The total number of tests performed and inconsistencies

Method	Test	Number of performed tests^1^	Number of detected inconsistencies
SIBCOUNT	Monozygotic twin	3	2
	Full-sib	114	4
	Half-sib	13568	107

SIBREL	Monozygotic twin	3	2
	Full-sib	114	4
	Half-sib	13568	110

Numbers of test per category (i.e. number of different sib relationships), and number of inconsistencies caused by the deleted animals

^1^The number of relationships that were tested per relationship category

### Type I and II error rates in permuted data

Type I and II error rates for SIBCOUNT and SIBREL were evaluated using the permuted data (Table 4). Both methods were preceded either by the deletion of inconsistent parent-offspring pairs (by PAR-OFF) or not. The type I error rate, i.e. the proportion of incorrectly deleted animals, was similar for both methods. Both methods showed a clear increase in the type I error rate when a high percentage of the pedigree was permuted (25%). When inconsistent parent-offspring pairs were not deleted, type I error rates were higher for both SIBREL and SIBCOUNT than when they were deleted prior to the test. Type I error rates for PAR-OFF also increased with increasing percentage of permuted pedigree, similar to SIBCOUNT and SIBREL.

**Table 4:** Type I and II error rates for the different methods

		PAR-OFF + SIB		SIB only	
		
Test	Permuted pedigree^1^	Type I	Type II	Type I	Type II
PAR-OFF	1%	0.0019	0.2970		
	10%	0.0210	0.3098		
	25%	0.0532	0.3409		
SIBCOUNT	1%	0.0001	0.2452	0.0003	0.0870
	10%	0.0055	0.0516	0.0252	0.0534
	25%	0.0269	0.0717	0.0920	0.0698
SIBREL	1%	0.0002	0.3132	0.0005	0.1421
	10%	0.0053	0.0981	0.0239	0.1295
	25%	0.0240	0.1127	0.0782	0.1256

Methods SIBCOUNT and SIBREL were either preceded by deleting animals causing parent-offspring inconsistencies (PAR-OFF) or not

^1^Pedigree data was permuted against genotype data for either 1%, 10%, or 25%

The type II error rate, i.e. the proportion of incorrectly non-deleted animals, was considerably higher for SIBREL than for SIBCOUNT. Not performing PAR-OFF first, substantially decreased the type II error rates for both SIBCOUNT and SIBREL when 1% of the data was permuted, while they remained similar when 25% of the data was permuted. Type II error rates were quite high when only 1% of the animals was permuted. And for PAR-OFF they were only slightly affected by the percentage of pedigree permuted.

To further gain insight on the performance of both tests, the absolute numbers of correctly deleted animals, and the number of type I and II errors were calculated (Table 5). The results revealed that PAR-OFF is the most important test to delete Mendelian inconsistencies, as is also the case in the overall raw data. Furthermore, Mendelian inconsistencies not deleted by the PAR-OFF test (type II errors), were largely deleted by the SIBCOUNT and SIBREL tests, as shown by the number of correctly deleted animals for both tests (Table 5). When omitting the PAR-OFF test, the total number of correctly deleted animals decreased slightly, but the decrease was greater for SIBREL than for SIBCOUNT (Table 5). At the same time, the total number of type I errors was somewhat different from that for SIBCOUNT or SIBREL alone, but no clear trend was observed in the differences. Clearly, the number of type II errors always increased when SIBCOUNT or SIBREL were performed alone, compared to when PAR-OFF was carried out first.

**Table 5:** Counts of numbers of deleted and non-dele ted animals

Test		Permuted pedigree^1^	Deleted	Type I	Type II
PAR-OFF + SIB	PAR-OFF	19.7	13.8	3.9	5.8
		206.8	142.7	38.3	64.1
		509.9	336.0	80.9	173.9
	SIBCOUNT	19.7	4.5	0.3	1.3
		206.8	57.0	10.1	7.3
		509.9	149.9	41.0	24.0
	SIBREL	19.7	4.0	0.4	1.8
		206.8	50.2	9.7	13.9
		509.9	136.2	36.5	37.7

	PAR-OFF +	19.7	18.3^2^	4.2^3^	1.3^4^
	SIBCOUNT	206.8	199.7	48.5	7.3
		509.9	485.9	121.9	24.0
	PAR-OFF +	19.7	17.9	4.2	1.8
	SIBREL	206.8	192.9	48.0	13.9
		509.9	472.2	117.5	37.7

SIB ONLY	SIBCOUNT	19.7	18.1	0.7	1.6
		206.8	195.7	46.0	11.1
		509.9	474.4	140.0	35.6
	SIBREL	19.7	17.0	1.0	2.8
		206.8	183.2	39.9	23.6
		509.9	446.0	119.0	64.0

Counts of correctly deleted animals (Deleted), and incorrectly deleted (Type I) and incorrectly non-deleted (Type II) animals

^1^Numbers of animals with permuted data, when pedigree data was permuted against genotype data for either 1%, 10%, or 25% of the animals. For SIBCOUNT and SIBREL after performing PAR-OFF, these numbers decrease to 5.9, 64.1, and 173.9, respectively (i.e. by subtracting the number of animals correctly deleted by PAR-OFF);

^2^sum of the number of animals deleted by PAR-OFF and SIBCOUNT (or SIBREL);

^3^sum of the type I errors of PAR-OFF and SIBCOUNT (or SIBREL);

^4^this figure is equal to the type II errors of SIBCOUNT (or SIBREL)

## Discussion

The objective of this paper was to present two tests that comprise a set of rules for fast identification of supposed sib pairs for which genotype and pedigree information are inconsistent, either based on counting opposing homozygotes (SIBCOUNT), or based on comparing pedigree and genomic relationships (SIBREL). Both algorithms performed similar in terms of type I error rate, but the SIBCOUNT algorithm performed better based on realized type II error rate. Both algorithms can be applied to edit SNP data that are obtained in an experiment or in a practical breeding program. Although we applied the methods to a dataset with relatively large (half-sib) families, both methods are expected to work equally well in populations with smaller (half-sib) families, judging from the clear distributions of the test statistics (Figure 1).

### Deleted animals

In the first steps, when removing parent-offspring inconsistencies (PAR-OFF), the derived threshold of 250 inconsistent SNP was close to the value of 200 used by Wiggans et al. [14] and more conservative than the cut-off value suggested from the distribution presented by Hayes [2] and the 2% of SNP used by Weller et al. [15]. Applying a 2% threshold is equivalent to 777 conflicting SNP in the present study. We used 250 SNP, but using 777 SNP as a threshold would not have changed the list of deleted bulls in our data.

Generally, SIBCOUNT and SIBREL performed similarly, as expected because the threshold for SIBREL was determined based on the corresponding count of opposing homozygotes (Figure 3). Our results clearly indicate that the proportion of animals with 'true' mismatches between pedigree and genotype data had an effect on type I and II error rates. With more true mismatches, the chance increases that an animal is deleted in error because of multiple relatives that can cause observed inconsistencies (type I error), while the animal that is causing the inconsistencies is not deleted (type II error). Type I error rates of the SIB tests, SIBCOUNT and SIBREL, were higher when they were not preceded by the PAR-OFF test. Type II error rates of the SIB tests were hardly affected by performing first the PAR-OFF test, when 10 or 25% of the data was permuted. However, when only 1% of the data was permuted, the type II error rate substantially increased for the SIB tests when they were preceded by PAR-OFF. Note that the type II error rates for the SIB tests are calculated as the proportion of animals with permuted pedigree that were not deleted. When the PAR-OFF is performed first, the type II error rate for the SIB tests includes animals with permuted pedigree data, which were deleted neither by the SIB test, nor by PAR-OFF. In this case, interpreting type I and II errors may be easier when comparing counts (Table 5) rather than rates (Table 4). In our data, the inflated type II error rates when using the SIB tests alone, indicate that the SIB tests alone can detect 94 to 99% of the Mendelian inconsistencies that would be detected if preceded by PAR-OFF. This means that the probability of detecting Mendelian inconsistencies for animals without any genotyped parent in our data was only slightly lower than for animals with at least one genotyped parent.

The type II error rate for PAR-OFF was between 0.297 and 0.341. Of all genotyped animals that were included to calculate type II error rates, 16% had no genotyped offspring or parent. Thus, those animals could not fail the PAR-OFF test and when permuted automatically contributed to the type II error rate. Therefore, it appears that the 'true' type II error rate for PAR-OFF for this data was ~0.14 to 0.18. Given the very high accuracy to detect inconsistent parent-offspring pairs, or to assign animals to parents using 50k SNP [2], these type II error rates appear to be high. The most likely reason is that, in our implementation, the PAR-OFF test deleted only one animal of an inconsistent pair when at least one of the animals was linked to more than one inconsistent parent-offspring pair. In some of these cases, an animal that actually caused the inconsistency may erroneously have been left in the data.

### Extensions to other relationships

In this study, we limited the detection of Mendelian inconsistencies to parent-offspring, half-sib or full-sib relationships. Both algorithms presented can, however, be extended to check for Mendelian inconsistencies for other relationships, such as half cousins, double cousins, or uncle-nephew relationships. Extensions with other relationships may help to avoid deleting animals incorrectly, when limited numbers of 1^st ^degree genotyped relatives are available. For the SIBCOUNT algorithm, the formula presented in this study can be extended to predict the expected numbers of opposing homozygotes between two animals with a particular relationship between them. For the SIBREL algorithm, general formulae that predict variances of a range of relationships have been presented by Hill and Weir [7].

Depending on the structure of the genotyped population, many animals may have e.g. several genotyped male ancestors that can be used instead. Wiggans et al. [1] presented a test for opposing homozygotes between animals and their maternal grandsires, using a threshold of 16% opposing homozygotes. In this case, the expectation of the number of opposing homozygotes, comparable to those for sib pairs, is greater than zero because the compared animals are more than one meiotic event apart in the pedigree. With an increasing number of meiotic events between two evaluated animals, both the expected number of opposing homozygotes and the variance of this expected number increases. This in turn implies that the performed test will lose power when the number of meiotic events between two compared animals increases, because the distribution of the tested parameter becomes wider and will more easily overlap with the distribution of this parameter in unrelated animals, just by chance. The same applies for sib relationships used in SIBREL e.g. [7]. Although both methods presented here can be expanded to test any type of relationship, the question is whether such tests can detect true inconsistencies without jeopardizing the type I and II error rates. As demonstrated in our study, comparing empirically the number of opposing homozygotes against the difference between genomic and pedigree-based relationships provides a straightforward way to get insight into which method is expected to be more accurate for a given class of relationships.

### Impact of (unidentified) inconsistencies

In our data, we removed ~10% of the genotyped animals, which is in line with reported values of misidentification in commercial herds of 5 to 13% [3,16-18]. An important reason to remove data with Mendelian inconsistencies is that such errors in the data may reduce the power of subsequent analyses. From earlier studies, it is known that removing records with incorrect pedigree information increases genetic gain and improves the accuracy of traditionally estimated breeding values [18-21]. The impact of unidentified inconsistencies depends on what is the objective when using these data. When the objective is to estimate SNP effects that will be used in genomic selection, it is important that the link between genotype and phenotype data is correct. If, however, predictions for parent average are also included in the predicted breeding values, then the link between pedigree and phenotype data should also be correct. Unidentified inconsistencies are also expected to affect results of genome-wide association studies. For instance, Huang et al. [22] reported that the power of genome-wide association studies decreases substantially when using imputed genotypes, even at low allelic imputation error rates. They postulated that to maintain power, the sample size should increase ~5% to 13% for each 1% increase in imputation error, in most populations.

## Conclusions

This study shows that tests for opposing homozygotes and comparison of genomic and pedigree-based relationships are powerful tools to detect sib pairs with inconsistent SNP and pedigree information. Counting the number of opposing homozygotes between pairs of sibs was slightly better at detecting inconsistent animals than comparing genomic and pedigree-based relationships, while both methods were equally likely to remove animals that in reality were consistent. Our results showed that tests to remove Mendelian inconsistencies between sibs should be preceded by a test for parent-offspring inconsistencies. These should be detected in two directions: 1) assuming that the pedigree is correct and test whether the SNP data of considered parent-offspring pairs is in agreement with the pedigree, and 2) assuming that the SNP data is correct and test whether the pedigree of considered parent-offspring pairs is in agreement with the SNP data.

## Competing interests

The authors declare that they have no competing interests.

## Authors' contributions

MPLC wrote the algorithms, performed the analyses and drafted the first version of the manuscript. HAM participated in discussions on the rules used in the described algorithm and critically contributed to the final version of the manuscript. JWMB performed the initial quality control steps on the SNP data and critically contributed to the final version of the manuscript. All authors read and approved the final manuscript.

## Appendix A

### Expected number of opposing homozygotes

In all derivations below, we assume that animals are not inbred. The probability that an inconsistency occurs on SNP locus *i *between any two animals is equal to the probability that the first animal is homozygous for the first allele and the second animal is homozygous for the second allele, or vice versa. This is hereafter referred to as the probability that two animals have opposing homozygous genotypes.

For two unrelated individuals in a population, given a frequency of *p_i _(q_i_) *of the first (second) allele at locus *i*, this probability is: $2p_{i}^{2}q_{i}^{2}$.

The number of expected opposing homozygotes across *n *loci for two unrelated animals, regardless whether any of the loci are linked to each other, therefore is: $\sum_{i=1}^{n}2p_{i}^{2}q_{i}^{2}$.

For two half-sibs in a population, the probability that they have opposing homozygous genotypes is equal to the probability that the common parent is heterozygous (2*p_i_q_i_*), both half-sibs receive different alleles from the common parent (2 × 1/2 × 1/2), and both half-sibs are homozygous, i.e. receive the same allele from the other parent as from the common parent (*p_i_q_i_*). This probability therefore is: $2p_{i}q_{i}2\frac{1}{4}p_{i}q_{i}=p_{i}^{2}q_{i}^{2}$.

The number of expected opposing homozygotes across *n *loci for two half-sibs is: $\sum_{i=1}^{n}p_{i}^{2}q_{i}^{2}$.

For two full-sibs in a population, the probability that they have opposing homozygous genotypes is equal to the probability that both parents are heterozygous $\left(4p_{i}^{2}q_{i}^{2}\right)$, both full-sibs receive different alleles from the first parent (2 × 1/2 × 1/2), and both sibs receive the same allele from the second parent as from the first parent (1/2 × 1/2). This probability therefore is: $4p_{i}^{2}q_{i}^{2}2\frac{1}{4}\frac{1}{4}=\frac{1}{2}p_{i}^{2}q_{i}^{2}$.

The number of expected opposing homozygotes across *n *loci for two full-sibs is: $\sum_{i=1}^{n}\frac{1}{2}p_{i}^{2}q_{i}^{2}$.

### Variance of expected number of opposing homozygotes

The variance of the expected number of opposing homozygous genotypes at locus *i*, can be derived by considering that this is the variance of a binomial variable: σ^2^(number of opposing homozygote genotypes) = *x*(1 - *x*)

where *x *is equal to the probability that two animals have opposing homozygous genotypes at locus *i*. This probability is derived above for pairs of unrelated animals, half-sibs and full-sibs. The variance of the expected number of opposing homozygotes across *n *loci, involves both the variance at each of the loci, but also the covariance between any pair of loci. The covariance of the expected number of opposing homozygotes between loci *i *and *j *is equal to the probability that two animals have opposing homozygotes both at locus *i *and *j*. Considering bi-allelic loci, with alleles 1 and 2 segregating at each locus, there are four possible haplotypes for loci *i *and *j*: 11, 12, 21, and 22, with probabilities *f_ij _*(11), *f_ij _*(12), *f_ij _*(21), and *f_ij _*(22). The probability that two unrelated animals have opposing homozygotes at both locus *i *and *j *is equal to the probability that the first (second) animal has two haplotypes 11 (22), or vice versa, plus the probability that the first (second) animal has two haplotypes 12 (21), or vice versa. The covariance in opposing homozygotes between locus *i *and *j *for two unrelated animals is equal to this probability, minus the probability that those animals are inconsistent between locus *i *and *j *due to chance rather than linkage disequilibrium between the loci (there are four scenarios, each with a probability of $p_{i}^{2}p_{j}^{2}q_{i}^{2}q_{j}^{2}$):

2$f_{ij}^{2}\left(11\right)f_{ij}^{2}\left(22\right)+2f_{ij}^{2}\left(12\right)f_{ij}^{2}\left(21\right)-4p_{i}^{2}p_{j}^{2}q_{i}^{2}q_{j}^{2}$.

For half-sibs, this covariance reduces by a factor 2, because animals need to receive a different haplotype from the common parent (which is only the case in two out of four possible scenarios). For full-sibs, this covariance reduces by a factor 4, because animals need to receive a different haplotype from the common parent (which is only the case in one out of four possible scenarios).

Ignoring recombination for closely linked loci, and assuming linkage equilibrium for non-linked loci, *f_ij _*(11), *f_ij _*(12), *f_ij _*(21), and *f_ij _*(22) are simply the frequency of the haplotypes in the population. For unlinked loci, i.e. loci with an (expected) recombination rate of 0.5, E(*f_ij _*(11)) = *p_i_p_j_*, E(*f_ij _*(22)) = *q_i_q_j_*, E(*f_ij _*(12)) = *p_i_q_j_*, E(*f_ij _*(21)) = *q_i_p_j_*, and therefore the expected covariance in opposing homozygotes between unlinked loci *i *and *j *is equal to zero. Therefore, we consider only covariances between pairs of loci that are located on the same chromosome.

Thus, the total variance of the number of opposing homozygotes between two unrelated animals is (for 1 chromosome with *n *loci):

$$\begin{array}{c} \overset{n}{\underset{i=1}{\sum }}2p_{i}^{2}q_{i}^{2}\left(1-2p_{i}^{2}q_{i}^{2}\right)+\\ \sum_{i=1}^{n}\sum_{\begin{array}{c} j=1,\\ j\neq i \end{array}}^{n}\left(f_{ij}^{2}\left(11\right)f_{ij}^{2}\left(22\right)+f_{ij}^{2}\left(12\right)f_{ij}^{2}\left(21\right)-2p_{i}^{2}p_{j}^{2}q_{i}^{2}q_{j}^{2}\right). \end{array}$$

The first term is the sum of the variances of all loci. The second term is the sum of all covariances between SNP on the same chromosome. This, after re-arrangement by grouping expressions that contain similar terms, reduces to:

$$\begin{array}{c} 2\overset{n}{\underset{i=1}{\sum }}p_{i}^{2}q_{i}^{2}+\\ 2\overset{n}{\underset{i=1}{\sum }}\overset{n}{\underset{j=i+1}{\sum }}\left(f_{ij}^{2}\left(11\right)f_{ij}^{2}\left(22\right)+f_{ij}^{2}\left(12\right)f_{ij}^{2}\left(21\right)\right)-\\ 4\sum_{i=1}^{n}\sum_{j=i}^{n}p_{i}^{2}p_{j}^{2}q_{i}^{2}q_{j}^{2}. \end{array}$$

And, realizing that *f_ii_*(11) = *p_i_*, *f_ii _*(22) = *q_i_*, and *f_ii _*(12) = *f_ii _*(21) = 0, the formula can be more generally written as:

$$\begin{array}{c} 2\overset{n}{\underset{i=1}{\sum }}\overset{n}{\underset{j=1i}{\sum }}\left(f_{ij}^{2}\left(11\right)f_{ij}^{2}\left(22\right)+f_{ij}^{2}\left(12\right)f_{ij}^{2}\left(21\right)\right)-\\ 4\sum_{i=1}^{n}\sum_{j=i}^{n}p_{i}^{2}p_{j}^{2}q_{i}^{2}q_{j}^{2}. \end{array}$$

Similarly, for two half-sibs, the total variance of the number of opposing homozygotes is

$$\begin{array}{c} \overset{n}{\underset{i=1}{\sum }}\overset{n}{\underset{j=1i}{\sum }}\left(f_{ij}^{2}\left(11\right)f_{ij}^{2}\left(22\right)+f_{ij}^{2}\left(12\right)f_{ij}^{2}\left(21\right)\right)-\\ 2\sum_{i=1}^{n}\sum_{j=i}^{n}p_{i}^{2}p_{j}^{2}q_{i}^{2}q_{j}^{2}. \end{array}$$

And, for two full-sibs, the total variance of the number of opposing homozygotes is

$$\begin{array}{c} \frac{1}{2}\overset{n}{\underset{i=1}{\sum }}\overset{n}{\underset{j=1i}{\sum }}\left(f_{ij}^{2}\left(11\right)f_{ij}^{2}\left(22\right)+f_{ij}^{2}\left(12\right)f_{ij}^{2}\left(21\right)\right)-\\ \sum_{i=1}^{n}\sum_{j=i}^{n}p_{i}^{2}p_{j}^{2}q_{i}^{2}q_{j}^{2}. \end{array}$$

The variance of the total number of opposing homozygous loci across the whole genome is the sum of the above variances for all chromosomes.

## Appendix B

The expected variance of half-sib relationships was calculated using the following formula [23]:

$V\left(P_{HS}\right)=\left(\frac{1}{128L^{2}}\right)\left[4L-v+\sum_{j=1}^{v}\exp\left(-4l_{j}\right)\right]$,

where *L *is the genome size, *v *is the number of chromosomes, and *l_j _*is the length of chromosome *j*. In this study we considered all 29 bovine autosomes. Therefore, *L *was considered to be 30.14 M, using the values for *l_j _*as reported by Ihara et al. [24]. For full-sib relationships, the expected variance is *2V*(*P_HS_*) [23].
